# Supplementary material for: Factors associated with preterm birth among mothers who gave birth at public Hospitals in Sidama regional state, Southeast Ethiopia: Unmatched case-control study
Source: PLoS One. 2022 Apr 20;17(4):e0265594. doi: 10.1371/journal.pone.0265594 (PMC9020679; doi:10.1371/journal.pone.0265594)
Supplement: S1 Appendix — (DOCX) [file pone.0265594.s001.docx]

# ANNEXS

## Annex I. English Version Information Sheet and Consent Form

Hawassa University College of Medicine and Health Science Department of Clinical Midwifery

Greeting;

Hello! My name is …………….I am currently a member of data collector in this study that
assess the determinants of preterm birth among mothers who gave birth in public hospitals of Sidama zone, southern Ethiopia. You were selected for the study by hoping that you will cooperate and respond genuinely. Kindly am requesting you to lend me your attention to explain you about the study. The study will be conducted through interviews and you are being asked for a little of your time, about 15-20 minutes, to help us in this study.

Your name will not be written in this form and will never be used in connection with any information you tell us. There is no possible risk associated with participating in this study except the time spent for responding to the questionnaire. All information given by you will be kept strictly confidential. Your participation is voluntary and you are not obligated to answer any question you do not wish to answer. If you feel discomfort with the question, it is your right to drop it any time you want. If you have questions regarding this study or would like to be informed of the results after its completion, please feel free to contact the principal investigator.

Address of the principal investigator:

Mr. Gossa Fetene; Cell phone: +251-947210135 E-mail: [Feteneg2119@gmail.com](mailto:Feteneg2119@gmail.com)

Would you willing to participate in this study?

1. Yes………………………………………………continue the next page
2. No……………………………………………….skip to the next participant

**Consent form;** Having been well explained and informed of the intentions and benefits of the study, I voluntarily consent to participate in this study.

Respondent’s signature_________________________________

Date of interview: _______________ Time started: _______ Time finished: _________

Interviewer Name_________________________Signature___________Date____________

Supervisor’s name ________________________ signature ________

## Annex II: English version questionnaires

A questionnaire to determine risk factors associated with preterm birth among mothers who gave birth in public hospitals of Sidama zone, southern Ethiopia, 2020 G.C.

1. Questionnaire ID number__________
2. Name of public hospital______________

Note: Encircle from the given option and write if any other idea or answer is given

**Part I.** Socio-demographic characteristics of mothers with their index neonates

| No. | Questions | Response | | Skip |
| --- | --- | --- | --- | --- |
| 101 | How old are you? | ………..(in year) | | Code |
| 102 | What is your current marital status? | 1. Single 2. Married 3. Others (specify)………….. | |  |
| 103 | What is your religion? | 1. Protestant 2. Orthodox 3. Muslim 4. Catholic 5. Others (specify) ……….. | |  |
| 104 | Where is your residence? | 1. Urban 2. Rural | |  |
| 105 | What is your educational status? | 1. No formal education 2. Primary school 3. Secondary school 4. College and above | |  |
| 106 | What is your current occupation? | 1. House wife 2. Gov’t employ 3. Farmer 4. Merchant 5. Daily labor 6. Student 7. NGO employ | |  |
| 107 | How much is your household monthly income in ETB? | | ……………(in Ethiopian birr) |  |
| 108 | How many members of family are there in your house? Please specify them in number | | …………………. |  |

**Part II:** Obstetric and medical related variables of mother

| No. | Questions | Response | Skip |
| --- | --- | --- | --- |
| 201 | How many pregnancies do you have? | ……….(pregnancies) |  |
| 202 | How many deliveries have you had? | ………...(deliveries) |  |
| 203 | By How many years’ interval you get this pregnancy? | 1…...in complete month/year  22. I don’t know |  |
| 204 | Did you have ANC follow up during your pregnancy for this Neonate? | 1. No 2. Yes | If no skip to Q208 |
| 205 | What was the gestational age of the fetus when you start ANC follow up? | 1………in weeks  22. I don’t remember |  |
| 206 | Where did you attend your ANC? | 1. Health center  2. Government Hospital  3. Private hospital  4. Private clinic  5. Other (specify) |  |
| 207 | How many times you visit health facilities for ANC? | …………(put in number)  22. I don’t remember |  |
| 208 | Have you told any danger symptoms of pregnancy during your ANC visits? | 1. No 2. Yes |  |
| 209 | Do you have History of preterm birth before this pregnancy? | 1. No 2. Yes |  |
| 210 | Did you have PIH during the pregnancy of this neonate? | 1. No 2. Yes |  |
| 211 | Did you have any bleeding during the pregnancy of this neonate?/ APH | 1. No 2. Yes |  |
| 212 | Have you told as you had any medical problems in the last 12 months related to this pregnancy? | 1. No 2. Yes | If no skip to Q214 |
| 213 | If your answer is yes for Q212, Which medical problems? (multiple answer is possible) | 1. Diabetes mellitus  2. Cardiac problem  3. Renal problem  4. Hypertension  5. Other (specify)… ……….. |  |
| 214 | Did you have any UTI/STI during the pregnancy of this neonate? | 1. No 2. Yes |  |
| 215 | Did you have premature rupture of membrane before onset of labor for this delivery? | 1. No 2. Yes |  |

Part III: Personal and social factors in the last 9 months.

| No. | Questions | Response | Skip |
| --- | --- | --- | --- |
| 301 | Have you chewed Khat in the last 9months? | 0. No 1. Yes | If no skip to Q303 |
| 302 | How often do you chew khat? | 1. Every day 2. At least once Per week 3. At least once Per fortnight 4. Occasionally 5. Not at all |  |
| 303 | Have you smoke cigarette in the last 9 month? | 1. No 1. Yes | If no skip to Q306 |
| 304 | How often do you smoke? | 1. Every day 2. At least once Per week 3. At least once Per fortnight 4. Occasionally 5. Not at all |  |
| 305 | How many cigarettes you smoke per day? | …………(in number) |  |
| 306 | Have you drink Alcohol in the last 9 months? | 0. No  1. Yes | If no skip to part IV |
| 307 | Which type of alcohol? | 1. Tella 2. Local areke  3. Beer 4. Wine 5. Teji  6.Other(specify) _____________ |  |
| 308 | How often you drink alcohol? | 1. Every day 2. At least once Per week 3. At least once Per fortnight 4. Occasionally 5. Not at all |  |

**Part IV:** Physical intimate partner violence against pregnant women

Now I would like to ask some questions about your relationship with your husband. Does/did your husband or other person ever done/act the following upon you within the last 9 months;

| 401 | Has he Push or shoved you, or shake your hands? | 0. No 1. Yes |
| --- | --- | --- |
| 402 | Has he slapped you or thrown something at you that could hurt you? | 0. No 1. Yes |
| 403 | Has he Punch or hit you with his fist, or twist your arm or with something that could hurt you? | 0. No 1. Yes |
| 404 | Has he Kick you, drag or beaten you? | 0. No 1. Yes |
| 405 | Has he tried to Attack you with a knife, gun, or other type of weapon? | 0. No 1. Yes |
| 406 | Have you scalded or burnt purposefully by your husband? | 0. No 1. Yes |
| 407 | Did the following ever happen because of something your husband did to you? | 1. You had bruises and aches 2. You had any injury or broken bone 3. Trauma to your abdomen/genitalia 4. Other/specify |
| 408 | Was there any of your property deliberately damaged when your husband used force against you even if the incident was not very serious? | 0. No 1. Yes 22. Can’t remember |
| 409 | How often any of the above offenses happen to you in the last nine months? | 1.Every day  2. At least once per wk 3. At least once/month 4. Once during pregnancy period 5. Not at all |
| 410 | Does any of the above listed violations were committed by other persons (friends, relatives, etc) upon you within the last 9 months? | 0. No 1. Yes |
| 411 | If yes Q410, who made this offenses? | 1. Mother in law 2. Father in law 3. Stranger 4. Others ………. |

**Part V**: Questions to be filled from medical records or by Measurement

| No. | | Questions | Source | Response |
| --- | --- | --- | --- | --- |
| 501 | | Gestational age of neonate at birth | Based on LNMP, or see card | …………weeks |
| 502 | | Sex of the neonate |  | 1. Male 2. Female |
| 503 | | Weight of mother at birth | Measure | ………… KG |
| 504 | | Height of mother at birth | Measure | ……....centimeter |
| 505 | | MUAC of mother | Measure | ………centimeter |
| 506 | | Current BP of the mother | Measure or see card | _____//____mmHg |
| 507 | Current sero status of the mother (HIV) | | Test or see from card | ..……….(R/NR) |

**Thanks a lot for your cooperation!!**
