# Supplementary material for: Factors associated with preterm birth among mothers who gave birth at public Hospitals in Sidama regional state, Southeast Ethiopia: Unmatched case-control study
Source: PLoS One. 2022 Apr 20;17(4):e0265594. doi: 10.1371/journal.pone.0265594 (PMC9020679; doi:10.1371/journal.pone.0265594)
Supplement: S2 Appendix — (DOCX) [file pone.0265594.s002.docx]

## Annex II: Amharic version of participant information sheet and consent form

ሀዋሳ ዩኒቨርስቲ ህክምና ና ጤና ሳይንስ ኮላጅ ሚድዋይፈሪ ትምህርት ክፍል

ለጥናት ተሳታፊዎች መረጃና የስምምነት ፎርም**/**ሰነድ

ጤና ይስጥልኝ! ስሜ---------------------ነው:: እዚህ የተገኘሁት ያለእድሜያቸው ከሚዎለዱ ህጻናት ጋር ተያያዥነት ያላቸውን ነገሮች ላይ ሳይንሳዊ ጥናት ለማድረግ ነው፡፡ ስለሆንም በቅድሚያ ስለ ጥናቱና ስለ እርስዎ ድርሻ እንደሚከተለው በማብራራት እጀምራለሁ፡፡

የጥናቱ ዓላማ፡**-** ያለእድሜያቸው ከሚዎለዱ ህጻናት ጋር ተያያዥነት ያላቸው ነገሮች ላይ ሳይንሳዊ ጥናት ለማድረግ ነው፡፡

ጥቅም፡- ትክክለኛ መረጃ መስጠት የህፃናትን ጤና ለማሻሻል እንደሚረዳ ማሳሰብ እወዳለሁ፡፡ እርስዎም የዚህ ጥናት አካል በመሆንዎ ከፍተኛ አስተዋፅኦ ያደርጋሉ፤ ከዚህ ውጪ ግን የገንዘብም ሆነ ሌላ የሚያገኙት ቀጥተኛ ጥቅም የለም፡፡

የጉዳት ሥጋት፡**-** የዚህ ጥናት አካል በመሆንዎ የሚደርስብዎት ምንም አይነት ጉዳት የለም፡፡ ምናልባት ጥያቄዎችን ለመመለስ ከ15-20 ደቂቃ ያክል ልወስድብዎት እችላለሁ፡፡

የጥናቱ መረጃ ምስጢራዊነት፡**-** እርስዎ የሚሰጡት ምላሽ ሁሉ በምስጢር የሚያዝና ለሌላ ሰው ተላልፎ የማይሰጥ ይሆናል፡መረጃው የሚያገለግለው ለዚህ ጥናት ብቻ ሲሆን ስምዎም ሆነ የስልክ ቁጥርዎ አይመዘገብም፡፡

የጥናቱ ተሳታፊዎች መብት፡**-** በጥናቱ የሚሳተፉት የእርስዎ ፈቃድ እስከሆነ ድረስ ብቻ ነው፡፡ በጥናቱ ሲሳተፉ
መመለስ የማይፈልጉትን ጥያቄ መዝለልና በፈለጉት ሰዓት ጥናቱን አቋርጠው የመውጣት መብትዎ የተጠበቀ ነው፡፡ ጥያቄ ካለዎት በየትኛውም ሰዓት ማንሳት ይችላሉ፡፡ እንግዲህ ከላይ ያነሳሁልዎትን ሀሳቦች ከግንዛቤ አስገብተው በጥናቱ ስለመሳተፍዎ ያለዎትን ውሳኔ ከዚህ ቀጥሎ ባለው ፎርም ላይ ይገልፁልኝ ዘንድ በትህትና እጠይቅዎታለሁ፡፡

**የስምምነት ውሳኔ መስጫ ፎርም**

ከዚህ በላይ ያለውን መረጃ አንብቤና በሚገባኝ ቋንቋ ተገልፆልኝ፤የጥናቱ ዓላማ፣ጥቅም፣ጉዳትና ምስጢራዊነት የተረዳሁ ሲሆን በጥናቱ ስለመሳተፌም ያለምንም ግፊት በራሴው ፍላጎት የሚከተለውን ወስኛለሁ፡፡

1. በጥናቱ ለመሳተፍ ወስኛለሁ (ወደ ሚቀጥለው ፎርም ይለፉ)

2. በጥናቱ ለመሳተፍ አልተስማማሁም (ሌላ የጥናት ተሳታፊ ጋር ይሂዱ)

የጥናት አድራጊው ስም፡**-** ጎሳ ፈጠነ አበበ

አድራሻ፡- ስልክ፡+251947210135 ኢሜል፡ [Feteneg2119@gmail.com](mailto:Feteneg2119@gmail.com) ይህ ቃለ መጠይቅ የተደረገበት ቀን---------የተጀመረበት ሰዓት----------- ያለቀበት ሰዓት------------የመረጃ ሰብሳቢው ስም-----------------ይህን ቃለ መጠይቅ የመረመረው የጥናቱ ተቆጣጣሪ ስም-----------------------ፊርማ--------

**ክፍል አንድ፦ የኢኮኖሚያዊ እና የማህበራዊ ሁኔታን የተመለከቱ ጥያቄዎች**

| መ.ቁ | ጥያቄዎች | የጥያቄዎች መለኪያዎች/ምላሾች | እለፍ |
| --- | --- | --- | --- |
| 101 | እድሜዎት በሙሉ ዓመት ስንት ነዉ? | ------------(በዓመት) |  |
| 102 | የጋብቻ ሁኔታ ምን ይመስላል? | 1. ያላገባች 2. ያገባች/አብረው የሚኖሩ/ 3. ሌላ(ይገለፅ) -------------- |  |
| 103 | የምን ሀይማኖት ተከታይ ነዎት? | 1. ፕሮቴስታንት  2. ኦርቶዶክስ 3. ሙስሊም 4. ካቶሊክ 5. ሌላ(ይገለፅ) -------------- |  |
| 104 | የት ነው የሚኖሩት? | 1. ከተማ 2. ገጠር |  |
| 105 | ከፍተኛ የትምህርት ደረጃሽ ስንት ነው? | 1.ት/ት አልተከታተልኩም 2. አንደኛ ደረጃ ት/ት 3. ሁለተኛ ደረጃ - መሰናዶ ት/ት  4. ከከፍተኛ የት/ት ተቋም የተመረቀ ወይም ከዛ በላይ |  |
| 106 | ስራሽ ምንድን ነዉ? | 1. የቤት እመቤት 2. የመንግስት ሰተራኛ 3. ግብርና 4. ነጋዴ 5. የቀን ሰራተኛ 6. ተማሪ 7. ሌላ(ይገለፅ) ------------- |  |
| 107 | በአማካኝ የቤተሰቡ የወር ገቢ ምን ያህል ነዉ? | ------------(በኢትዮጵያ ብር ይገለጽ) |  |
| 108 | የቤተሰብ ብዛት ስንት ነው?በቁጥር ያስቀምጡ | ……………………… |  |

**ክፍል-ሁለት፡ እርግዝናንና ወሊድን እንዲሁም አጠቃላይ የጤና ሁኔታን የተመለከቱ ጥያቄዎች**

| ተ/ቁ | ጥያቄዎች | መለኪያ | እለፍ |
| --- | --- | --- | --- |
| 201 | እስከ አሁን ስንት ጊዜ አርግዘው ያዉቃሉ? (የአሁኑን ጨምሮ) | -------------(በቁጠር ይገለጽ) |  |
| 202 | እስከ አሁን ስንት ልጆች ወልደዋል? (የአሁኑን ጨምሮ) | -------------(በቁጠር ይገለጽ) |  |
| 203 | ይህን ህጻን በምን ያህል ጊዜ ልዩነት ነው የወለዱት? (ከዚህ በፊት ከነበረው እርግዝና ጋር ሲነፃፀር) | --------------(በወራት ይገለጽ)  22. አላስታውስም |  |
| 204 | ለዚህ ህጻን የቅድመ_ወሊድ ክትትል አድርገው ነበር? | 0. የለም  1. አዎ | የለም ከሆነ ወደ 208 ይለፉ |
| 205 | የቅድመ_ወሊድ ክትትል ማድረግ የጀመሩት እርግዝናዎ ስንት ወር ሲሆነው ነበር? | 1. ------- (በወር ይገለጽ) 22. አላስታውስም |  |
| 206 | የቅድመ ወሊድ ክትትል የጀመሩት የት ነበር? | 1. ጤና ኬላ 2. ጤና ጣቢያ 3. የመንግስት ሆሰፒታል 4. የግል ሆሰፒታል 5. መንግስታዊ ያልሆነ ክሊኒክ 6. ሌላ(ይገለፅ)---------- |  |
| 207 | ለቅድመ_ወሊድ ክትትል ብቻ ስንት ጊዜ ሀኪም ቤት ሄደዋል? | --------------(በቁጥር)  22. አላስታውስም |  |
| 208 | በእርግዝና ክትትሉ ወቅት አደገኛ ስለሚባሉ ምልክቶች የምክር አገልግሎት አግኝተው ነበር? | 0. የለም  1. አዎ |  |

| 209 | ከዚህ እርግዝና በፊት በነበሩት እርግዝናዎች 9 ወር ሳይሞላ መወለድ ገጥሞት ነበር? | 0. የለም  1. አዎ |  |
| --- | --- | --- | --- |
| 210 | ከዚህ እርግዝና ጋር ተያይዞ የተፈጠረ የደም ግፊት ነበረብዎት? | 0. የለም  1. አዎ |  |
| 211 | በዚህ እርግዝና ወቅት የደም መፍሰስ አጋጥሞት ነበር? | 0. የለም  1. አዎ |  |
| 212 | ባለፉት 9 ወራት ውስጥ ከእርግዝና ጋር የተያያዙ የጤና እክሎች/ችግሮች/ገጥሞዎት ነበር? | 0. የለም  1. አዎ | የለም ከሆነ ወደ 214 ይለፉ |
| 213 | ለጥያቄ 208 መልሱ አዎ ከሆነ; የትኞቹ የጤና እክሎች/ችግሮች/ አጋጠመዎት? (ከአንድ በላይ መመለስ ይቻላል) | 1. ስኳር በሽታ 2. የኩላሊት በሽታ 3. የልብ በሽታ 4. የደም ግፊት 5. ሌላ(ይገላፅ)----- |  |
| 214 | በዚህ እርግዝና ወቅት የአባላዘር በሽታ አሞት ያውቅ ነበር? | 0. የለም  1. አዎ |  |
| 215 | በዚህ እርግዝና ወቅት ከምጥ በፊት የእንሽርት ውሃ ፈሶ ነበር? | 0. የለም  1. አዎ |  |

**ክፍል ሶስት፡ ከአኗኗር ዘይቤና የግል ባህርያት ጋር የተያያዙ ጥያቄዎች**

| መ.ቁ | ጥያቄዎች | መልስ | ይለፍ |
| --- | --- | --- | --- |
| 301 | ባለፉት 9 ወራት ውስጥ ጫት ቅመው ያውቃሉ? | 0. የለም  1. አዎ | የለም ከሆነ ወደ 303 ይለፉ |
| 302 | ባለፉት 9 ወራት ውስጥ ስንት ጊዜ ጫት ቅመው ያውቃሉ? | 1.በየቀኑ 2. በሳምንት አንድ ጊዜና ከዛ በላይ 3. በሁለት ሳምንት አንድ ጊዜና ከዛ በላይ  4.አልፎ አልፎ 5. በፍፁም ቅሚ አላቅም |  |
| 303 | ባለፉት 9 ወራት ውስጥ ሲጋራ አጭሰዋል? | 0. የለም  1. አዎ | የለም ከሆነ ወደ 306 ይለፉ |
| 304 | ባለፉት 9 ወራት ውስጥ በየስንት ጊዜው ሲጋራ ያጨሳሉ? | 1.በየቀኑ 2. በሳምንት አንድ ጊዜና ከዛ በላይ 3. በሁለት ሳምንት አንድ ጊዜና ከዛ በላይ  4.አልፎ አልፎ 5. በፍፁም አጭሸ አላቅ |  |
| 305 | በቀን ምን ያህል ሲጋራ ያጨሳሉ? | ---------------- (በቁጠር ይገለፅ) |  |
| 306 | በህይወት ዘመንዎ አልኮል ጠጥተዉ ያውቃሉ? | 0. የለም  1. አዎ | የለም ከሆነ ወደ ክፍል 4 ይለፉ |
| 307 | የትኞችን የአልኮል ዓይነት ይጠቀማሉ? (ከኣንድ በላይ መልስ ይቻላል) | 1. ጠላ 2. አረ 3. ቢራ 4. ወይን 5. ጠጅ 6. ሌላ(ይገለፅ)------- |  |
| 308 | ባለፉት 9 ወራት ውስጥ በየስንት ጊዜው አልኮል ይጠጣሉ? | 1. በየቀኑ 2. በሳምንት አንድ ጊዜና ከዛ በላይ 3. በሁለት ሳምንት አንድ ጊዜና ከዛ በላይ 4. አልፎ አልፎ 5. በፍፁም ጠጥቼ አላቅም |  |

**ክፍል-አራት፡ በነፍሰ ጡር እናቶች ላይ የሚፈፀሙ አካላዊ ጥቃቶችን የተመለከቱ ጥያቄዎች**

| **ተ/ቁ** | **ጥያቄዎች** | **መልስ** |
| --- | --- | --- |
| 401 | በእርግዝናዎ ጊዜ በባለቤትዎ በሀይል ተገፍትረው ያውቃል? | 0. የለም 1. አዎ |
| 402 | በባለቤትዎ በዚህ እርግዝና በጥፊ መመታት ወይም የሆነ ነገር ተወርውሮብዎት ያውቃል? | 0. የለም 1. አዎ |
| 403 | በባለቤትዎ ባለፉት 9 ወራት ውስጥ በቦክስ መመታት፣ የመጠምዘዝ ወይም በሌላ ነገር መቶዎት ያውቃል? | 0. የለም 1. አዎ |
| 404 | ባለቤትዎ ቢላዎ፣ ጠብመንጃ፣ ወይም ሌላ የጦር መሳሪያ አደጋ መዞብዎት ያውቃል? | 0. የለም 1. አዎ |
| 405 | ባለቤትዎ ሆን ብለው የማቃጠል ወይም የማነቅ አደጋ አድርሰውብዎት ያውቃሉ? | 0. የለም 1. አዎ |
| 406 | ባለቤትዎ በርግጫ/በካልቾ መቶዎት፡ ደብድቦዎት ያውቃል? | 0. የለም 1. አዎ |
| 407 | በባለቤትዎ አካላዊ ጥቃት ምክንያት ከሚከተሉት ውስጥ የደረሰብዎት ጉዳት አለ? (ከአንድ በላይ መልስ ይቻላል) | 1. የሰውነት መቁሰልና ማበጥ 2. የአጥንት መሰበር አደጋ 3. የመራቢያ አካል ጉዳት 4. የሆድ እቃ ጉዳት 5. ሌላ(ይገለፅ)----- |
| 408 | ባለፉት 9 ወራት ውስጥ ባለቤትዎ ከላይ የተጠቀሱትን የአካላዊ ጥቃቶች በየስንት ጊዜው ይፈፅሙበዎት ነበር? | 1. በየቀኑ 2. ቢያንስ በሳምንት ውስጥ አንዴ 3. ቢያንስ በወር ውስጥ አንዴ 4. ቢያንስ በዚህ 9 ወር አንዴ 5. ምንም አልተፈፀመብኝም |
| 409 | ከላይ ከተጠቀሱት የጥቃት ዓይነቶች ውስጥ በሌላ ሰው ማለትም በጓደኛ፣ በዘመድ፣በእንግዳና በሌሎች በእርስዎ ላይ ባለፉት 9 ወራት የተፈፀሙ አሉ? | 0. የለም 1. አዎ |
| 410 | ለጥያቄ 409 መልስዎ አዎ ከሆነ, ማነው ጥቃት የፈጸመብዎት? | 1. ሴት አማቴ 2. ወንድ አማቴ 3. በእንግዳ ሰው 4. ሌላ (ይገለጽ) ____ |

**ክፍል 5: በመለካት ወይም ከካርድ ላይ የሚሞሉ ጥያቄዎች**

| **ተ/ቁ** | **ጥያቄ** | **ምንጭ** |  |
| --- | --- | --- | --- |
| 501 | ህፃኑ ሲወለድ የነበረው የእርግዝና እድሜ | ከ LMP በመነሳት ተቆጥሮ ፧ ከካርድ ላይ የሀኪም ዲያግኖሲስ/ የአልትራሳውድ ውጤት በማየት የሚሞላ | ________(በሳምንትይገለጽ) |
| 502 | የህፃኑ ዖታ | በማየት የሚሞላ | ……….…(ወንድ/ሴት) |
| 503 | እናትየዋ ክብደት | ተለክቶ የሚሞላ | ______(በኪሎ ግራም ይገለጽ) |
| 504 | የእናትየዋ ቁመት | ተለክቶ የሚሞላ | ____ (በሴንቲ ሜትር ይገለጽ) |
| 505 | እናቱ ህፃኑ ሲወለድ የነበራት MUAC ስንት ነበር? | ተለክቶ የሚሞላ | _____(በሴንቲ ሜትር ይገለጽ) |
| 506 | የእናቲቱ የግፊት መጠን | ተለክቶ / ካርድ ታይቶ የሚሞላ | ___// __ (በሚሜ ሜርኩሪ) |
| 507 | የእናቲቱ የኤች ኣይቪ ሁኔታ | ተለክቶ / ካርድ ታይቶ የሚሞላ | ______ (ኔጋቲቨ/ፖዘቲቭ ) |

**ስለ ትብብሮዎ ከልብ አመሰግናለሁ!!**
