# Supplementary material for: Factors associated with preterm birth among mothers who gave birth at public Hospitals in Sidama regional state, Southeast Ethiopia: Unmatched case-control study
Source: PLoS One. 2022 Apr 20;17(4):e0265594. doi: 10.1371/journal.pone.0265594 (PMC9020679; doi:10.1371/journal.pone.0265594)
Supplement: S3 Appendix — (DOCX) [file pone.0265594.s003.docx]

## Annex III: Sidaamu Afiini Qixxabbinot Qeqaanote Mashshalaqe Amadino Sumuu Yaate Forme

Hawassi universite xagisattena fayyimate sience ilishiishshate ros goli buuxate beeqaano taje sumuu yaate forme

Keere keeshitn! Su’ma’yya---------------------yinannie:: koye afammoomohu kalaa gosa fexeneha riqiwaten ikkanna፣ isino hawassi univeriste ilishiishshate rosi goli layink degree roso rosate aana afamanno፡፡ konni rosira biso ikkino xiinxallo asinannita ikkitanna kunino diru illikinni ilantanno qaaquule ledo amadamino coye xinxalteeti ፡፡ikkinohura balanxe xiinxalote daafira xinxallona itera noohe qeecha aannino garin xawisate hanafeemo ፡፡

**Xiinxallote kora** ፡**-** diru iilikkin ilanttano qaaquulei ledo amadantinorich aana siensaawe xiinxallo assateeti ፡፡

**Horo፡-**buuxantinno taje aate qaaquulu fayyima woyyeesate kaa’lannota buuxisiisate baxxeemmo ፡፡ Atino tenne xiinxallo biso ikatenni jawaata qeechcha assatto ፤ kunni gobaanni kayiinni wxehono ikko wole afi’nay suwasho horo dino ፡፡

**Gawajamate huluulo**፡**- tenne xiinxalo biso ikootohura assatorira mitte gawajono dino**፡፡ miteeke xa”muwa dawarate 15-20 daqiiq geeshsha adhanoha ikkanno

**Xiinxalote mashalaqe maafudase**፡**- ati aato/ta tumo baalanti maafuda dinbannitanna wolu mannira saayinse diuyiinanni** ፡ mashalaqe horonsi’nanihu tenne xiinxalora calla ikkinohura su’mano ikko blibilu kiironno diborreesinani ፡፡

**Xiinxalote beqaanch qoosso**፡**-** xiinxalote beqaanchi is fajo ikkiro calaati፡፡

xiinxalote beqaanchchci dawara hasirannoki xa’mo dawarikkini agure sa’ana hasirino yannara xiinxalo agure fulas qooso agarantionote:;xa’mo heedhusiro aye sa’aterano xa’ma dandaano:;
፡፡ konni daafira aleeni kayiinsummo hedo hedote gidora eessatenni xinxalote beeqate gumolokki aane noo forme aana xawisatoe gede shaqilunni xa’mireemmo::

**Sumuu yaate gumulo aate form**

konni aleenni noo mashalaqe nababenna leelanno qaalinni xawiseie፤ xiinxalote kora ፣horose፣gojona maafudasi huwatinoha ikkanna xiinxalote beeqatenna mite xiiwonno nookiha umis fajonni aananoha gumuloomo

1. Xiinxalote beeqate gumuloommo (aantino formera sai)

2. xiinxalote beeqate sumuu diyoomo (wole xiinxalote beqaanch ledo sai)

Xiinxallo assanohusu’ma:- Gosa Fexene Abebe

Heeranno base ፡- blbilu kiiro፡+251947210135 emaile፡ [Feteneg2119@gmail.com](mailto:Feteneg2119@gmail.com) tenne qalu xa’mo assinooni barra --------hannfoonni yanna ----------- gumuloonni yanna ------------taje gamba assinohu su’ma ---------tenne qaalu xa’mo qorqorannohu su’ma--------..mallate--------

**Gafa mite፦ miinjuna dagoomitete akata lainohun shiqino xa’mo**

| **kiiro** | **Xa’mo** | **Xa’muwa bikinaani /dwaran** | Sai |
| --- | --- | --- | --- |
| 101 | Diriki me’eho? | ------------(diruni) |  |
| 102 | Galtete ledo noorichch maa lawanno? | 1. Adhanitinokita 2. adhantinota/ ledo heedhanota / 3. wole(xawis) ------------ |  |
| 103 | May ama’nno harunsatta? | 1.benxe  2. ortodokise 3. musilime 4. kaatolike 5. wole(xawis) -------------- |  |
| 104 | Heeranno base? | 1. quchchuma 2. Baadiyye |  |
| 105 | Rosu deeri me’eho | 1.roso dirosoomo 2. 8 kifile 3. 12 - kifile  4. kolejete geeshshanna hakuyi alee |  |
| 106 | Loosiki maati? | 1. Loosu dinoe 2. Mangistete loseemma 3. baatto 4. dadalo 5. barru looso 6. roso 7. wole(xawis) ------------- |  |
| 107 | Maatekira aganu eo me’eho? | ------------tophiyu birrin xawis) |  |

**Gafa 2፡ godowatenna ilate hattono xaphooma fayyimate akata laino xa’muwa**

| kiiro | **Xa’mo** |  | **Sai** |
| --- | --- | --- | --- |
| 201 | Xaageeshsha me’e hige godowoota? (xaahano lendeena ) | ------------(kiirotenni xawisi) |  |
| 202 | Xaa geeshshame’e ooso illoota? (xaaha lendeena) | ------------(kiirotenni xawis) |  |
| 203 | Konne qaaqo me’ek dir badooshshin iloota? (albiidihu ledo hewiisiisiro) | -----------(aganatenni xawis)  22. diqaageema |  |
| 204 | Konne qaaqo ilita wote balaxote buuxo asiroota? | 0. Dee’ni  1. ee | Dino ikkiro 208 sai |
| 205 | Balaxote ila buuxirate hanafootahu godwitankuuni meeki aganiraat? | 1. ------- (aganuni xawis) 2. dibuuxooma |  |
| 206 | Balaxote ilate buuxo hanafootahu hiikoot? | 1. fayyimate keelira 2. fayyimate xaawira 3.mangistete hosopitaalera 4.gilete hosipitaalera 5.mangistaawe ikkitinokki kilikera 6.wole xawisi)---------- |  |
| 207 | Baalaxote ilate buuxo harunso me’e hige haroota? | --------------kiiroten)  2.diafooma |  |
| 208 | godobe heedhe hakimete buuxo assirata wote busha malaataati yinanir horo afidhe egenoota? | 0.dee’ni  1.ee |  |

| 209 | xaa godowa kirra albaanni noo godowakki 9agannini ila xaade egentinohe? | 0. dee’ni  1. ee |  |
| --- | --- | --- | --- |
| 210 | tenne godowaki ledo amadante it kalaqantino mundeete xiiwo nooni? | 0.dee’in  1.ee |  |
| 211 | tenne godowakira yannara mundeete du’namma xaadinohenni? | 0.dee’n  1.ee |  |
| 212 | sau 9 aganara gido godowate ledo amadante fayyimate qarri xaadinohenni? | 0. dee’n  1.eee | Nookiha ikkiro 214 sai |
| 213 | Xa’mo 208 daworo ee ikituro hiiku fayyimate qarri xaadinohe mittu aleen qola dandiinanni | 1. Sukaarete xisso 2.mulu xiso  3. Wodanu xiso 4.mundeete xiiwo  5.wole xawis- |  |
| 214 | Tenne godowa yannara taraawanno xiba leele egenino? | 0.dee’n  1. ee |  |
| 215 | tenne godowa yannara gamete albaanni du’nammanori fulinoni? | 0.dee’ni  1. ee |  |

**Gafa 3 heeshshote akatana gilete akati ledo amadantino xa’muwa**

| **kiiro** | **xa’mo** | Dawaro | **Sai** |
| --- | --- | --- | --- |
| 301 | Sau 9 agana gido caate qamae egenoota? | 0.de’ni  1.ee | Tumo dino ikiro 303 sai |
| 302 | Sau 9 aganna gido me’e hige caate horonsidhe egenoota ? | 1.barru baala  2. Lamalate mite hige  3. Lame lamara mite hig  4.sae sae 5.horontanni |  |
| 303 | Sau 9 aganna giddo sigaara horonsiroota? | 0. dee’n  1. ee | Dee’ni ikiro 306 sai |
| 304 | Sau 9 aganna giddo sigaara me’e hige horonsiroota? | 1barra baala. 2.lamalate mite hige  3. Lame lamala mite hige nna hakuyi alee  4sae sae. 5. Horonta diegenooma |  |
| 305 | Barrunni me’e sigaara horonsirata? | ---------------- (kiirotenni xawis) |  |
| 306 | Heeshshoki diro alcohole age egenoota? | 0.dee’ni  1.ee | Dee’n ikiro 4 sai |
| 307 | Hitee alcoholete dana horonsirata mite dawaro aleeni dandiinanni? | 1. farsho 2. era 3.biira 4.woyiine 5. xaje 6. Wole xawis------- |  |
| 308 | Sau 9 aganna gido me’e hige alkoole agata? | 1. barra baala  2.lamalate mite higenna hakuyi alee 3. Lame lamala gido mite higenna hakuyi ale 4.sae sae 5.horontanni |  |

**GAFA 4:- GODOWINN NOO AMA AANA IKKITANNO BISATE GOJO LAINNOHUNNI XA’MO**

| **kiiro** | **xa’muwa** | **dawaro** |
| --- | --- | --- |
| 401 | godowate yannara minaankikin wolqate xiwaante egenoota? | 0. Dee’ni 1ee |
| 402 | Minaanikin xa godowakira angatenni gana woyiwolere ole egenino? | 0. Dee’ni 1. ee |
| 403 | Minaanikin sau9 aganna gido tuntu’motenni gana shakishe woy wole coyin gane egeninohe? | 0. Dee’ni 1.ee |
| 404 | Minaanik bilawunni;qawettenni woyi wolu olu uduunichin qaraiilishshe egeninohe? | 0.dee’ni 1.ee |
| 405 | Minaanikki samiyee giirate qara woyi qalxate dano iilishshe egeninohe? | 0.dee’ni 1.ee |
| 406 | Minaaniki lekatenni gane egeninohe ? | 0.dee’ni 1.ee |
| 407 | Minaanikin bisu gawajo korkoaatin aananori giddo iilinohe gawajo nooni?mite aleeni dawro dandiinanni | 1. mannimate madirana darshsha  2. Miqichu hiiqama gawajo 3.sirote bissa gawajo  4. Godowu gido gawajo 5.wole xawis----- |
| 408 | Sau 9 agannara gido minaaniki aleeni xawinsoonni bissu gawajo me’e hige iilishshinohe? | 1. barra baala  2.ajana lamalatenni mite hige  3. Ajanna aganunni mit hige  4. Ajana konni aganni mitege  5.mitoreno |
| 409 | Aleenni xawinsoonni gawajote dani giddo wolu manni yaano jaala ;fiixa ;wosinchona wolootu ate aana sau 9aganara iilishinor nooni | 0. Dee’ni 1. ee |
| 410 | Xa’mo 409 dawaro ee ikkituro ayeeti gawajo iilishinohu | 1. meyaa amaati 2.labaaho  3. wosinchoho  4. wole(xawis) ____ |

**GAFA 5:BIKATENI WOY KAARDETE AANINI WONSHINANI XA’MUWA**

| **kiiro** | **Xa’mo** | **Buicho** |  |
| --- | --- | --- | --- |
| 501 | Qaqu Ilami Wote Magee Yannini Ilam? | LMP Ka’ne Kiriro;Kaardete Aaninihakime Altrasoundeteguma Lae Wonshitannote | ________(Lamalate Xawis |
| 502 | Qaaqu Koo/Tee | La’atenni Wonshinaniho | ______(Labaaha/Meyaata/) |
| 503 | Amate Kiilo | Bikine Wonshinanniho | ____(K/Graametenn Xawi) |
| 504 | Amate Hoja | Bikine Wonshinaniho | ____(Me’e Meetirexawis?) |
| 505 | Qaaqu Ilami Wote Noosi MUAC Me’ete? | Bikine Wonshinaniho | ______ (Me’e Meetire Xawinsanni ) |
| 506 | Amate Mundeete Xiiwo Me’ete | Bikine Kaardete Aanini Wonshinanniho | ___// __ (Mm/Merkureni) |
| 507 | Amate Yeetayv Akata | Bikine Kaardete Aannini Wonshinaaniho | ______ (-Ve/+Ve ) |

**Kaa’lote Daafira Wodannuni Galateeemo!!**
