# Supplementary material for: Factors associated with preterm birth among mothers who gave birth at public Hospitals in Sidama regional state, Southeast Ethiopia: Unmatched case-control study
Source: PLoS One. 2022 Apr 20;17(4):e0265594. doi: 10.1371/journal.pone.0265594 (PMC9020679; doi:10.1371/journal.pone.0265594)
Supplement: Attachment — Submitted filename: Response to Reviewers.docx [file pone.0265594.s005.docx]

**Author response to reviewers**

**Dear Editor and Reviewers,**

Thank you very much for your email dated 14^th^ August 2021 incorporating the insight of the editor and reviewer’s comments. On behalf of all authors, I express our gratitude to you for the critical and constructive review that has led to the great improvement of our paper entitled ***“*Factors associated with preterm birth among mothers who gave birth at public Hospitals in Sidama regional state, Southeast Ethiopia: Unmatched case-control study”**. We have carefully reviewed the comments given from the editor and all the two reviewers and revised the manuscript accordingly. Our responses are given in a point-by-point manner below for respective editor and reviewers' comments.

**Version 1:** PONE-D-21-10270

**Date:** 8/27/2021

**Academic editor comments and a respective author response**

**Editor comment 1:** Please ensure that your manuscript meets PLOS ONE's style requirements, including those for file naming. The PLOS ONE style templates can be found at [**https://journals.plos.org/plosone/s/file?id=wjVg/PLOSOne_formatting_sample_main_body.pdf**](https://journals.plos.org/plosone/s/file?id=wjVg/PLOSOne_formatting_sample_main_body.pdf) **and** [**https://journals.plos.org/plosone/s/file?id=ba62/PLOSOne_ formatting_ sample_ title_authors_affiliations.pdf**](https://journals.plos.org/plosone/s/file?id=ba62/PLOSOne_%20formatting_%20sample_%20title_authors_affiliations.pdf)

**Author response:** Thanks very much for this comment. The whole parts of the manuscript was updated as per the PLOSE ONE style templates.

**Editor comment 2:** Please include additional information regarding the survey or questionnaire used in the study and ensure that you have provided sufficient details that others could replicate the analyses. For instance, if you developed a questionnaire as part of this study and it is not under a copyright more restrictive than CC-BY, please include a copy, in both the original language and English, as Supporting Information.  If the original language is written in non-Latin characters, for example Amharic, Chinese, or Korean, please use a file format that ensures these characters are visible.

**Author response 2:** Thanks very much for this comment. Amharic and Sidaamu Afoo versions of the questionnaires used in this manuscript were attached in the supporting informations ***(S2 Appendix, and S3 Appendix)****.*

**Editor comment 3:** In your Data Availability statement, you have not specified where the minimal data set underlying the results described in your manuscript can be found. PLOS defines a study's minimal data set as the underlying data used to reach the conclusions drawn in the manuscript and any additional data required to replicate the reported study findings in their entirety. All PLOS journals require that the minimal data set be made fully available. For more information about our data policy, please see <http://journals.plos.org/plosone/s/data-availability>.

**Author response 3:** Thanks very much for this insightful comment. The underlying data used in the manuscript was attached in the supporting informations **(*SAV. 1*)**.

**Editor comments 4:** Your ethics statement should only appear in the Methods section of your manuscript. If your ethics statement is written in any section besides the Methods, please move it to the Methods section and delete it from any other section. Please ensure that your ethics statement is included in your manuscript, as the ethics statement entered into the online submission form will not be published alongside your manuscript.

**Author response 4:** Thanks very much for this comment. The ethical declaration statement had moved to the method section based on the editor's comment.

**Editor comment 5:** Please include a separate caption for each figure in your manuscript.

**Author response 5:** Thanks very much for this comment. The caption had been given for the figure.

**Editor comment 6:** Please upload a copy of Figure 2, to which you refer in your text on page 5. If the figure is no longer to be included as part of the submission please remove all references to it within the text.

**Author response 6:** Thanks a lot for this critical and very insightful comment. Figure 2 had no longer available and it was removed.

**Editor comment 7:** Please include captions for your Supporting Information files at the end of your manuscript, and update any in-text citations to match accordingly. Please see our Supporting Information guidelines for more information:  [http://journals.plos.org/plosone/s/ supporting-information](http://journals.plos.org/plosone/s/%20supporting-information).

**Author comment 7:** Thanks very much for your insightful comment. The caption had been given for all supporting information files included at the end of the manuscript.

**Reviewer #1 comments and an author response**

Reviewer #1: In this manuscript, the authors investigated factors associated with preterm birth at public hospitals in Sidama regional state, Southeast Ethiopia, 2020. They found that the risk of PTB is increased in women living in rural areas, with no ANC follow-up, who have UTI, PIH, chronic medical problems, low birth spacing, and suffer with physical violence during pregnancy.
The study is well designed, the results are clear, the manuscript is well written and clinical significance is high. But following comments/questions should be addressed prior to acceptance.
**Reviewer comment 1**: Materials and Methods: The authors described that the definition of PTB is delivery before 37 completed gestational weeks, but the control group of this study consisted of women who delivered at 28-36 weeks of gestation. Is there any reason for excluding extremely early PTB at less than 28 weeks of gestation?

**Author response 1:** Thanks very much for this question. Since the study was done in Ethiopia, preterm birth is defined as the delivery of the neonate after 28 weeks of gestation and before 37 completed weeks. If the delivery ended before 28 weeks of gestational age, it was considered as abortion according to the countries guideline (Ethiopia-MOH-Obstetrics-Protocol-2020, page 172). Available at; <https://www.scribd.com/document/505962352/Ethiopia-MOH-Obstetrics-Protocol-2020>

**Reviewer comment 2:** Materials and Methods: Calculating gestational age correctly is most important for determining cases and controls (preterm and term birth). In this study, gestational age was confirmed by LMP or early ultrasound records. However, calculating gestational age by LMP may not be correct in many women (especially when their periods were irregular or very long), and recall bias may occur. Since, the antenatal check was not done in 30% and 13% of the cases and controls, there may be misclassification bias. This issue should be included in the Discussion section.

**Author response 2:** Thanks very much for your insightful comment. It was considered as a limitation of the study which introduced recall bias and explained in the last sentence of the discussion as directed by the reviewer (*See Page* *19*).

**Reviewer comment 3:** Discussion: The authors stated that the reason for the higher risk of PTB in rural residents may be hard physical works like farming. Is there any evidence for this explanation?

**Author response 3:** Thanks a lot for your critical question. In Ethiopia, many women who resided in rural areas are exposed to hard physical works like farming, fetching water, gathering wood. A study conducted by Van Beukering et. al. showed that women who were exposed to hard physical works during pregnancy had higher odds of preterm birth than those who were not exposed.

*Van Beukering, MDM Van Melick, MJGJ Mol, BW Frings-Dresen, et al. Physically demanding work and preterm delivery: a systematic review and meta-analysis. International archives of occupational and environmental health. 2014; 87(8)*

**Reviewer comment 4:** The strengths and weaknesses of this study should be discussed in the Discussion section.

**Author response 4:** Thanks very much for this comment. The strength and weaknesses of the study had been discussed in the last sentence of the discussion section as commented by the reviewer (*See page 19*).

Minor comments ;

**Reviewer comment #1:** Introduction, first line: what is ‘tremidinous’? Is it ‘tremendous’?

**Author response 1:** Thanks a lot for your critical insight and for correcting our wording error. Yes, it is ‘tremendous’. It was revised and corrected in the sentence.
**Reviewer comment #2**: What is MDG 4? The full term of the abbreviations should be presented.

**Author response 2:** Thanks a lot for your comment. The term MDG 4 is an abbreviation and stands for “Millineum Development Goal 4”. It was revised and stated in the full term (*See page 4, line 7* ).
**Reviewer comment #3**: Figure 2 is not shown.

**Author response 3**: Thanks a lot for your insightful comment. Figure 2 had no longer available and it was removed.

**Reviewer #2 comments and an author response:**

Reviewer #2: Review PONE-D-21-10270
Factors Associated with Preterm Birth among Mothers Who Gave Birth at Public Hospitals in Sidama Regional State, South East Ethiopia: Unmatched Case Control Study
This study investigates risk factors of preterm birth in the Sidama region of south east Ethiopia with one of the highest prevalence of preterm births in the world. The aim is to provide insight into risk factors of preterm birth in the region to enable comparison with other nations research on preterm birth.

**Major**
**Reviewer comment 1:** Have cases and controls been selected to obtain equal frequencies of male / female neonate sex in cases and controls? The equal male frequency of 58.5% in cases and controls seems to be somewhat unexpected if controls are drawn by random. Could the authors please see if this is due to method or comment on this?

**Author response 1:** Thanks very much for your comment. The equal frequencies of male / female neonate sex in cases and controls were due to chance. We used an unmatched case-control study and cases were selected consecutively and for each case, two controls were taken randomly (*See page 6*).
The discussion could be improved by including something on covariation of risk factors and elaborate on how combinations of these could be less or more important in this region of high prevalence of preterm birth especially compared to in other countries. See several points in discussion below.

**Minor**

**Reviewer comment 1:**

Abstract
In sentence informing about software applied it should be EpiData without hyphen:
“Data were entered using Epi-data version 3.1 and exported to SPSS version 20 for analysis.

**Author response 1:** Thanks very much for your comment. It was revised and corrected as commented by the reviewer.

**Reviewer comment 2:
Introduction**
At page 4 (top section) the authors mentioned some other studies in the field, are any of those possible to include as sources?
“But, in Ethiopia, few studies were conducted on risk factors associated with preterm birth and showed contradicting findings across different geographical settings and different periods.”
**Author response 2:** Thanks a lot for your comment. It was revised and referenced as commented by the reviewer (*See page 4, line 15*).

**Reviewer comment 3:**
**Results**
Obstetrics related characteristics
Gravidity is categorized into equal to or above 5 and less than 5 which is less informative than primiparous compared to multiparous, is it enough available data to get information on primiparous in the study?
**Author response 3:** Thanks for your insightful comment. Previous studies showed that the risk of preterm birth among grand multiparas was more likely as compared to primipara as well as multipara. Also, we haven’t had enough available data to get information on primiparous in the study to compare the odds of preterm birth among primipara with multipara as well as grand multipara.

**Reviewer comment 4:** Variable name should be ‘first antenatal care started’ instead of ‘fist antenatal care started”.

**Author response 4:** Thanks very much for your comment. It was revised and corrected as commented by the reviewer.
**Reviewer comment 5:** Based on frequency of pregnancy-induced hypertension have these pregnancies been assessed for preeclampsia?
**Author response 5:** Thanks a lot for your critical comment. In this research, pregnancy-induced hypertension includes gestational hypertension, pre-eclampsia, and Eclampsia.
**Reviewer comment 6: Pre-existing medical problems related characteristics**
On page 10 the authors use the category ‘chronic medical problems’ without describing in further detail. “Chronic medical problems were diagnosed among 39(28.9%) of the cases and 26(9.6%) of the controls group.”
Is it possible to include some examples of which chronic conditions or medical problems that would classify into this variable?

**Author response 6:** Thanks a lot for this comment. Chronic medical problems were assessed by asking a woman about having been diagnosed with diabetes mellitus, cardiac problem, chronic renal problem, chronic hypertension, chronic liver disease, and others.
**Reviewer comment 7:** In table 3 describing maternal pre-existing medical problems, height has an above and below 150 cm threshold, could the authors describe this variable more, is in relation short stature from to malnutrition?

**Author response 7:** Thanks for this critical comment. The classification of the height of the mother was made based on the previous studies. Previous studies showed that the odds of preterm birth among short stature (height less than 150 cm) mothers were more likely as compared to those mothers whose height ≥ 150 cm long. Even though, short stature had a clinical association with malnutrition, in our study it had no statistical association.

**Reviewer comment 8: Fetal related characteristics**
See major concern above on equal neonatal male frequency in cases and controls.

**Author response 8:** Thanks very much for this comment. The equal distribution of male to female neonates was happened due to chance. The selection of cases and controls were discussed under the section sampling technique (*See page 6*).
**Reviewer comment 9: Factors associated with preterm birth**
Table 5, variable pregnancy-induced has a misprint in AOR 2.870(1..519, 5.424)

**Author response 9:** Thanks very much for your comment. The AOR of the variable pregnancy-induced hypertension was revised and corrected as AOR 2.870(1.519, 5.424).

**Reviewer comment 10:**
**Discussion**
The authors include comparable studies in discussion. Include in the discussion how the results compare to published data also where they deviate. Are there some results that should be expected to associate with preterm birth that show less association than expected in these results?
Please discuss if the results indicate a less prominent role of malnutrition as a risk factor of preterm birth in the region.
For instance, in the data material, the frequency of birth defects seems high. Could many of these be associated to malnutrition (like cleft palate) in a sense that birth defects from malnutrition, even high, is not a prominent risk factors of preterm births in the region? Could this be interpreted in relation to many of the maternal pre-existing medical problems that are malnutrition related and not prominent drivers of preterm birth in the dataset. Could the authors comment on malnutrition and nutritional aspects in relation to preterm birth in the region from the data in the study?

**Author response 10:** Thanks very much for your comment. The variable nutritional status of the mothers had no statistically significant association with preterm birth according to our study, but had a statistically significant association in previously conducted study. And it was revised and disicussed in the discussion section (*See page 20, paragraph 1*).

**Reviewer comment 11:** If possible, could the authors elaborate on probable covariation of some of the included risk factors? Is it possible to observe any interaction between rural residency and physical violence or between rural residency and use of antenatal care? Considering rural facilities on sanitation and access to clean water, could possibly rural residency and factors as PPROM and UTI show interaction? Place of ANC follow up seems to indicate some association to rural residence as well.

**Author response 11:** Thanks very much for this comment. The correlation between each independent variables was assessed to test multicollinearity with a variance inflation factor (VIF) with a maximum VIF of 1.158 (*See page 9, line 15-16*).
